# Exploring the multi-level impacts of a youth-led comprehensive sexuality education model in Madagascar using Human-centered Design methods

**DOI:** 10.1371/journal.pone.0297106

**Published:** 2024-04-10

**Authors:** Sara E. Baumann, Laura Leeson, Mihaja Raonivololona, Jessica G. Burke

**Affiliations:** 1 University of Pittsburgh, Department of Behavioral and Community Health Sciences, Pittsburgh, PA, United States of America; 2 Projet Jeune Leader, Antsirabe, Madagasgar; City University of New York, UNITED STATES

## Abstract

Comprehensive sexuality education (CSE) is recognized as a critical tool for addressing sexuality and reproductive health challenges among adolescents. However, little is known about the broader impacts of CSE on populations beyond adolescents, such as schools, families, and communities. This study explores multi-level impacts of an innovative CSE program in Madagascar, which employs young adult CSE educators to teach a three-year curriculum in government middle schools across the country. The two-phased study embraced a participatory approach and qualitative Human-centered Design (HCD) methods. In phase 1, 90 school principals and administrators representing 45 schools participated in HCD workshops, which were held in six regional cities. Phase 2 took place one year later, which included 50 principals from partner schools, and focused on expanding and validating findings from phase 1. From the perspective of school principals and administrators, the results indicate several areas in which CSE programming is having spill-over effects, beyond direct adolescent student sexuality knowledge and behaviors. In the case of this youth-led model in Madagascar, the program has impacted the lives of students (e.g., increased academic motivation and confidence), their parents (e.g., strengthened family relationships and increased parental involvement in schools), their schools (e.g., increased perceived value of schools and teacher effectiveness), their communities (e.g., increased community connections), and impacted broader structural issues (e.g., improved equity and access to resources such as menstrual pads). While not all impacts of the CSE program were perceived as positive, the findings uncovered opportunities for targeting investments and refining CSE programming to maximize positive impacts at family, school, and community levels.

## Introduction

### Comprehensive Sexuality Education (CSE) in schools

CSE is an evidence-based mechanism that equips young people with information regarding sexual and reproductive health and the tools needed for developing and maintaining healthy, non-violent relationships [1, 2]. Schools, where adolescents spend most of their time, provide an avenue to directly reach youths with CSE interventions that are replicable and scalable [3]. Evaluations of in-school CSE programs have found that they offer cost-effective means to provide essential information and resources in a structured learning environment, as well as offer important linkages to health and social services [4]. In high-income countries, there is strong evidence that school-based CSE can build understanding of gender expectations and gender-based oppression; reduce dating and intimate partner violence; improve skills for maintaining healthy relationships; and improve feelings of self-efficacy and safety in relation to child abuse [5]. In-school CSE is especially needed in low-income countries (LICs) where youth, particularly girls, face serious threats to their well-being from violence, childhood marriage, early and unintended pregnancy, and school dropout [6].

Despite a salient need, CSE is a long way from being adopted and maintained in LIC school systems [7]. This stems from deep-rooted misconceptions that CSE will encourage sexual activity and corrupt youth [8, 9]. Indeed, CSE has traditionally been conceptualized as a sexually transmitted infection (STI) and pregnancy prevention intervention and assessed for its impact on sexual and reproductive health [10, 11]. Ivanova et al. [2020] found the health-related outcomes (i.e., pregnancy and sexually transmitted infection [STI] rates) are the most common metrics of CSE effectiveness [12]. With CSE long characterized by youth sexuality–often viewed as a sensitive and morally-imbued topic–political will and commitment for CSE is still poor, especially within resource-constrained education systems with competing priorities [13, 14].

### Impacts of CSE beyond pregnancy and STI prevention

While CSE has traditionally been conceptualized as a STI and pregnancy prevention intervention [10, 11], recent global discourse on CSE has moved beyond individual-level sexual health knowledge and behavior change, with evaluation efforts following close behind. Pioneering scholarship by Venwesenbeeck [2016] suggested that “Effective implementation of sexuality education may benefit teacher-student relationships in the classroom, parent-child communication, community norms and school social climate” [15]. Additional research conducted in Mexico demonstrated that a gender-transformative CSE program, delivered by specialized and highly trained educators, supported prevention of intimate partner violence among youth [16]. In another example from rural United States, CSE programming via the LiFT program led to significant improvements in parent-child communication about sexuality [17]. Furthermore, a 2020 systematic review highlighted that CSE can improve students’ social-emotional learning skills; however, none of the included studies were from a LIC [5].

Exploration and articulation of change mechanisms underlying CSE that work to prevent violence, improve teacher-student relationships, and shift family and community norms are needed to effectively advocate for and scale up evidence-based CSE interventions. This is especially critical in constrained LIC education systems with competing priorities [7]. Evidence regarding the complex ways in which CSE may be impacting lives and communities beyond primary health-related outcomes is also needed to improve CSE’s perceived need and fit within existing education systems, especially among government decision-makers [7, 18].

### Adolescent health and education in Madagascar

Located 250 miles to the East of the African Continent, Madagascar is the fourth largest island in the world with a population of 26 million, close to half of whom are younger than 15 years of age. Adolescents, especially girls, in Madagascar experience some of the poorest health and educational outcomes in the world. Early pregnancy is one of the most pervasive and persistent issues; the adolescent fertility rate increased from 148 in 2009 to 163 in 2012 and endures at 151 as of 2018 [19]. This is one of the highest rates in the Eastern and Southern Africa region; one in three Malagasy girls will become a mother before the age of 18.

The high and disparate rates of early childbearing in Madagascar are influenced by several factors, such as child marriage (41% of girls are married before their 18^th^ birthday, and 1 in 10 before they turn 15) and early sexual intercourse (20% of girls aged 15–19 years had their first sexual intercourse before the age of 15) [19]. Additionally, prevailing social norms in a traditionally male-dominated society often put women and girls at risk for human rights abuses, mistreatment, and gender-based violence that increases risk for early pregnancy. A staggering 10% of Malagasy girls aged 15 to 19 have experienced sexual violence from a partner [20]. Furthermore, alarming levels of violence are experienced during adolescence. At the household level, 86% of Malagasy children have experienced some form of violent discipline, 63% have experienced physical punishment, and 10% have experienced severe physical punishment [20]. Notably, 58% of youth have experienced violence at school [20]. This evidence suggests that adolescence is a critical period for intervening to address violence, child marriage, and early childbearing.

Complicating the situation in this resource-limited setting, is the fact that public resources for health and education services are limited due to Madagascar’s multifaceted political and economic environment. Within the health sector, coverage of sexual and reproductive health programs is restricted, with contraceptive prevalence (any modern method) at 46.1%, and the unmet need for family planning (any modern method) at 20% [21]. Further, service providers rarely possess the required skills to care for youth and adolescent health needs. Madagascar’s education system is also under considerable constraints, with spending at just $19 USD per student per year [22]. These structural challenges perpetuate academic underachievement, where less than a quarter of primary students have basic reading skills, just 7% have basic math skills, and school dropout rates are alarming (i.e., only one out of every four students that enter middle school will finish) [19].

Recognizing early pregnancy as a public health priority, Madagascar is a signatory of the Eastern and Southern Africa (ESA) Ministerial Commitment to Institutionalization of Comprehensive Sexuality Education (CSE) [23]. However, this 2013 commitment is far from being operationalized. Given the pressing need to address the determinants of early childbearing in this complex setting, there is ongoing need to develop and test innovative CSE programs and measure their multi-faceted, long-term impacts [29].

While there are numerous social and structural forces influencing health and decision-making of youth in Madagascar, including but not limited to global markets, social norms, and societal expectations, the Projet Jeune Leader (PJL) CSE program seeks to fill gaps in addressing student knowledge, and reduce burdens on schools. It offers an integrated curriculum that can be embedded into the free hours in students’ schedules, building upon the existing education structures and providing an entry point for improving adolescent health in Madagascar.

### Human-centered Design research

Human-centered Design (HCD) is an approach that aims to engage diverse stakeholders in exploring needs and creating people-focused solutions that consider individual experiences, motivations, beliefs, and desires [24–27]. Praised for its ability to facilitate the development of culturally relevant and practical solutions, HCD also increases the likelihood of community uptake and program sustainability [24, 28]. It offers participatory and creative tools to facilitate the design process with users and is flexible and iterative [29], which allows for the approach to be applied differently depending on the context and key challenges. It encourages teams to be responsive to project needs as they evolve [24].

HCD stems primarily from business and technology sectors [24], and has been long embraced by the design and architecture fields to address diverse challenges [30]. However, HCD is on the rise in public and global health, where it has been used to define community problems, design interventions, evaluate program impacts, increase the usability of health solutions, and more [29, 30, 31]. In fact, numerous studies have found that HCD is particularly well-suited to address global health challenges [28, 32–35]. Teams across several regions and continents have utilized HCD to tackle diverse health-related issues, such as enhancing HIV care, addressing noncommunicable diseases, and combating violence against women [28, 33, 36]. Studies have involved a broad spectrum of stakeholders, including community members, governmental leaders, policymakers, health experts, and funders [24]. By embracing HCD in global health programming and research, teams can optimize stakeholder engagement, tailor approaches to meet community needs [33], and embrace new ways of thinking and problem solving through cycles of creative and iterative solution development, testing, and monitoring [24].

#### Projet Jeune Leader’s (PJL) CSE program

PJL is a youth-centered organization in Madagascar whose mission is to transform schools and communities through comprehensive sexuality education (CSE), ensuring all youth reach their full potential. The program aims to ensure young adolescents, especially girls, in rural Madagascar live, learn, and grow up in environments supporting their health and well-being. To achieve this goal, the program targets key constructs of health and well-being, specifically mutually respectful and equitable relationships, healthy behaviors, healthy relationships with parents and school leaders, and linking survivors of violence and students with medical concerns to services. Program inputs directly address these constructs via structured, youth-centered, and time-tabled CSE classes, as well as extracurricular enrichment opportunities for students, parents, and school leader engagement, as well as provision of referral services.

PJL’s innovative sexuality education model is a three-year intra-curricular program designed specifically for students in grades 6, 7, and 8 (ages 10–15 years), taught to boys and girls together. The program is led by highly trained and specialized young adult CSE educators, who are integrated into government middle schools to teach the multi-year curriculum during students’ normal timetables. Educators strategically fill unused learning hours due to a lack of teachers and classrooms in Madagascar’s education system. The program currently targets rural areas of Madagascar with the poorest adolescent reproductive health outcomes, but PJL continues to scale up the program throughout the country.

The curriculum follows a fun, participatory, and interactive learning approach proven to facilitate quality learning by engaging learners and encouraging them to personalize information. The curriculum is also built upon a gender-transformative approach, which emphasizes substantive discussions on gender, power, and identity to challenge harmful norms and practices. As such, lessons throughout the curriculum involve: encouraging critical awareness about unhealthy, rigid, and harmful gender and sexual norms; questioning the impacts of harmful, inequitable gender norms and making explicit the advantages of changing them; and building on positive existing norms around non-violence, healthy relationships, and non-discrimination. The curriculum also adopts an empowerment approach that promotes essential life skills such as young adolescents’ confidence and self-efficacy, effective communication, maintaining healthy interpersonal relationships, and informed decision-making that serve as protective factors from violence and unwanted pregnancy. The curriculum consists of 27 dedicated, age-segmented scripted lessons that follow a logical sequential approach for the 81 total lessons throughout middle school (27 lessons per year, for three years). Each lesson lasts approximately one hour, and educators typically teach one lesson per grade per week throughout the school year. For students, their PJL courses are a regular part of their school week and middle school experience. The CSE lessons are required but students are not graded. Interspersed with essential sexual and reproductive health topics–puberty, anatomy, menstruation, fertility–are leadership lessons. Students learn assertive communication skills, methods to resolve conflicts, how to manage emotions, the importance and how-to of setting goals, and how to think critically and solve difficult problems they will face throughout their lives.

When educators are not teaching, they offer additional services as part of the CSE model: personalized counseling sessions, healthcare and social services referrals, extracurricular and enrichment activities, and workshops for parents. When students have a question or concern related to their health or well-being, educators refer students to local healthcare professionals. Educators are supported to refer students who experience abuse or violence to PJL’s existing partners at the Regional Ministry of Population and Social Services. The CSE curriculum, personalized support, and positive modeling all work together to improve knowledge on how and where to report incidents and aim to break the ‘culture of silence’ on violence. Educators also conduct participatory, and mixed-sex groups of extracurricular/enrichment activities for their students. Depending on the school, these dedicated activities can be timetabled into students’ schedules. Educators have access to a set of PJL’s Health and Leadership magazines to use with their students, but they are also encouraged to use their creativity and local resources to conduct additional fun and safe activities. Finally, educators are responsible for delivering a half-day parents’ workshop on positive parenting and effective communication with adolescent children.

Overall, the multi-faceted and youth-centered CSE program utilizes positive modeling (for adolescents, parents, and other teachers), a gender-transformative and empowerment approach, personalized information and support, and fun, participatory pedagogy delivered in a structured and high exposure intervention.

PJL has been implementing the CSE model since 2013, and pre-post school year evidence with comparison schools suggests that the program model has had a positive effect on adolescent students’ knowledge, attitudes, self-efficacy, and behavioral intentions toward sexual and reproductive health and rights and gender equality [37]. However, there remains a gap in understanding the multi-faceted and long-term impacts of the CSE model. This article explores the complex ways in which CSE programming may be affecting adolescent students, families, schools, and communities, from the perspective of school administrators and principals.

## Materials and methods

We used HCD methods within a two-phased approach to facilitate the exchange of ideas among program leadership and implementers to ultimately discover the widespread impacts of the CSE approach on the lives of students, parents, educators, and communities and prioritize areas for action.

### HCD framework and approach

Our study is informed by the IDEO’s framework for HCD, a leading design company that has utilized HCD techniques to design innovative solutions from topics ranging from reducing food waste, to digitally managing diabetes [38]. According to IDEO, HCD has three distinct phases: 1) discovery, 2) ideation, and 3) implementation [30, 38]. In the discovery phase, HCD allows teams to learn how to better understand communities and their needs through deep listening, keen observation, and empathy building. The ideation phase consists of generating numerous ideas and opportunities to test and refine. Finally, in the implementation stage, the ideas from the previous two stages are tested in the real world and refined to maximize their impact [39]. A key strength of HCD is that the tools can be applied to a wide range of issues in an endless number of combinations to meet project goals. In this manuscript we focus on the discovery phase, and applied HCD tools that allowed us to capture unique perspectives from a range of individuals. Future activities building upon this work will harness HCD tools designed for ideation and implementation.

Our study is also informed by the Theory of Change for Guiding the Integration of Human-Centered Design into Global Health Programming, which articulates how HCD can strengthen global health processes and problem solving by working collaboratively with providers, managers, and funders; future work will aim to incorporate end-users [24]. Finally, we utilized the Luma Institute design system and associated “recipes,” to select a combination of data collection methods based on our end goals. The Luma Institute system includes 36 design methods (i.e., ingredients) that can be mixed and matched in various combinations (i.e., recipes) to meet a project’s unique needs [40].

### Data collection

Data was collected using two consecutive methods from the Luma Institute system, specifically “affinity clustering” and “visualize the vote” (Table 1) [39]. Data collection occurred in two phases.

**Table 1:** Human-centered Design (HCD) activities, descriptions, and outputs.

HCD Activities	Description of Activities and Associated Steps	Outputs
***Phase 1–90 participants representing 45 schools***		
Brainstorming and Affinity Clustering	1. Participants individually brainstormed ideas in response to the first prompt and wrote as many ideas as they could think of on post-it notes, capturing one idea per post-it note. 2. The facilitator guided the participants in “Affinity Clustering,” in which the participants read their ideas from the post-it notes aloud and placed them on the wall in clusters based on perceived similarities and differences. 3. This process continued until all participants shared all their ideas. 4. The facilitator guided the group in a discussion to generate a summative label for each cluster, by asking probing questions (e.g., What do these items have in common? What would you call this cluster to summarize its contents?). 5. The facilitator guided the participants in a discussion to draw comparisons and identify potential relationships between clusters and items in clusters. The facilitator drew arrows and wrote notes to illustrate the relationships described by participants.	• Exhaustive list of related ideas that address the research question • Cluster names of key issues/themes related to the research question • Description of relationships between key themes
***Phase 2–50 participants representing 50 schools***		
Synthesis Presentation	1. The facilitator shared a brief presentation reminding participants of the original brainstorming and affinity clustering activity and explained the methods to those who were not in attendance in phase 1 (e.g., there were 5 new schools added between phase 1 and phase 2).	• Overview presentation of initial findings
Visualize the Vote	1. The clusters identified in phase 1 were written on flipchart paper and posted around the conference room for “Visualize the Vote.” Each flipchart had three labeled spaces for participating principals to post their vote to depending on if they had seen that impact in their school. The three options were: “Have seen,” “Have not seen,” “Don’t know.” 2. The participants completed a “gallery walk” to review and vote on each cluster (Fig 1). 3. The facilitator led the group in a discussion to provide participants an opportunity to share reflections about the clusters of ideas that they have seen in their schools. Given the large number of participants, the discussion focused on clusters that had the most, observable “have seen” votes. The facilitator asked principals to provide explanations for clusters impacted by CSE program. 4. After three (at most) principals had shared their thoughts, the facilitator moved on to discussing the next cluster. 5. Finally, the facilitator asked principals to reflect individually on which impact of the program is most important to them. Participants were asked to reflect silently for 30 seconds. 6. The principles were then asked to move across the room to stand next to the cluster they deemed as most important.	• Tally of the number of schools in which the cluster themes are experienced. • Identification of priority areas by principals for targeting CSE efforts.

**Fig 1:**
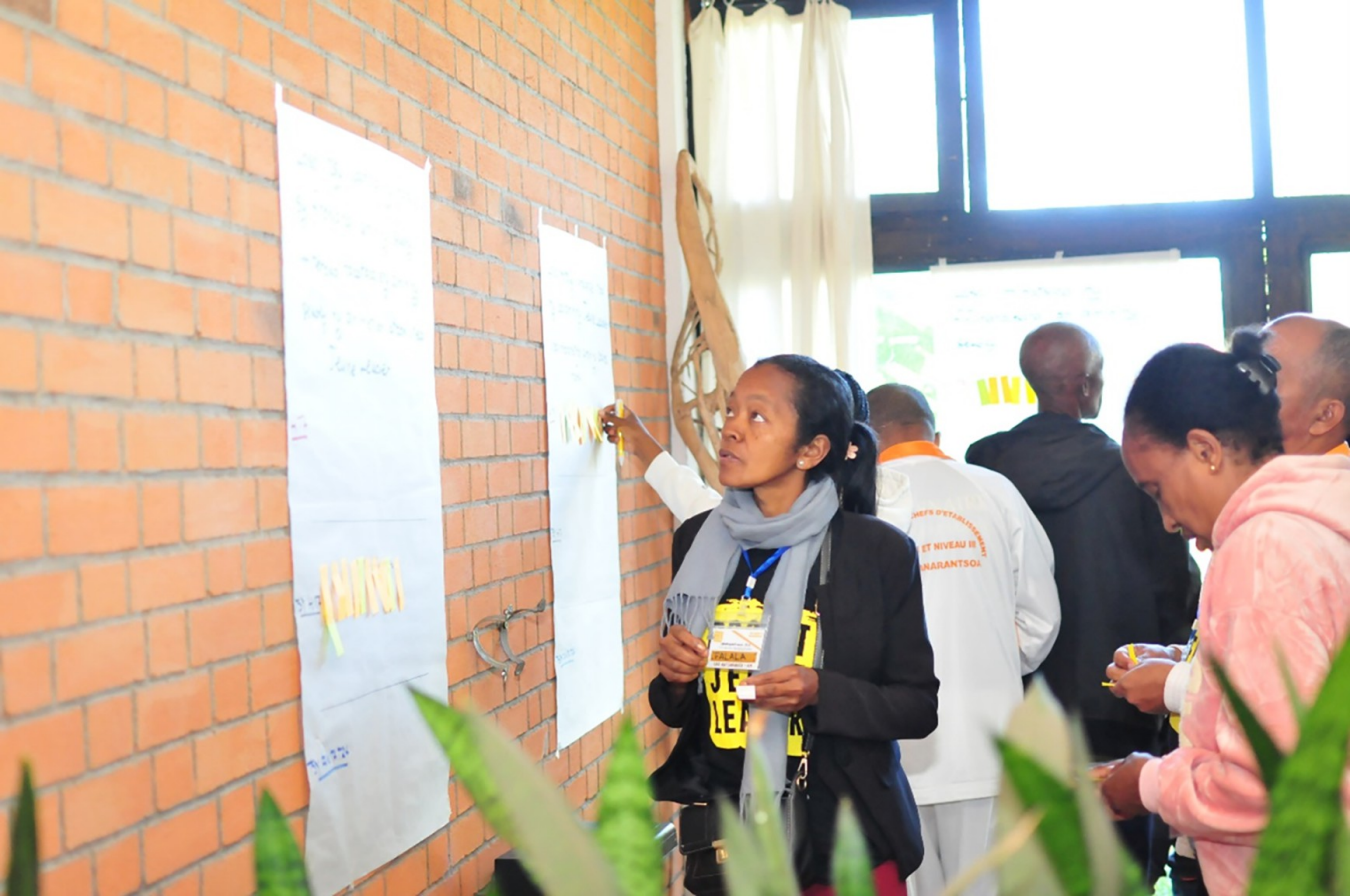
A participating principal reads the cluster label. The flipchart had three sections to vote on: "Have seen (this impact)," "Have not seen," and "Don’t know".

### Phase 1

Data were collected in June 2021 during seven workshops held in six regional cities in Madagascar. Principals and one other member of the school were invited from each of PJL’s 45 partner schools during the 2020–2021 school year. Principals had the discretion to invite second representatives, which was often the person who worked most closely with or alongside the CSE Educator. If the principal could not attend, then they assigned someone to attend in their place. In total, 90 school administrators participated, which included principals, vice principals, and school monitors representing 45 schools, both urban and rural.

School principals are among PJL’s most important local stakeholders. Principals sign an official agreement with PJL every school year, communicate regularly with PJL’s program team, and participate in monitoring and supporting PJL’s CSE educators. Therefore, it was critical to include their perspectives in this study.

**Fig 2:**
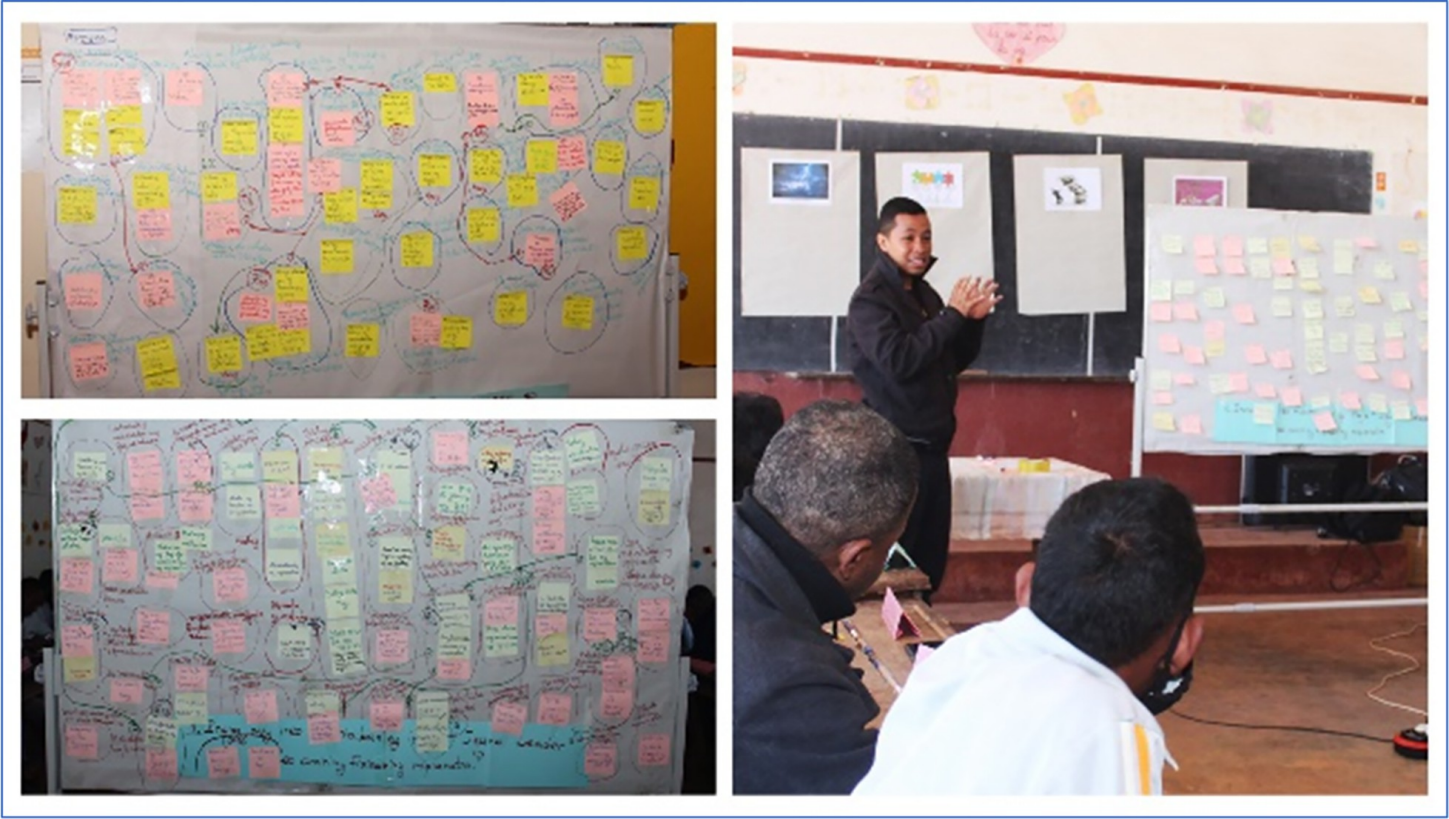
The facilitator led participants in a brainstorming and affinity clustering exercise regarding the research prompts.

Workshops were facilitated by PJL’s Monitoring and Evaluation Manager and lasted between three and four hours. Workshops were held in PJLs regional offices or rented working space (i.e., not in schools), and discussions were facilitated in the local language, Malagasy (Fig 2). Additional PJL staff members in attendance took detailed notes, which they then cross-referenced with the audio recordings of the discussions as needed to accurately fill a standardized data entry sheet in Microsoft Excel. The final data entry sheet was translated into English for qualitative analysis.

At the workshops, participants responded to two research prompts:

1. What are the different effects CSE programming has on students’ lives?
2. What other effects does the CSE programming have in the school or in the community?

In brief, participants brainstormed responses to the prompts individually on post-it notes, then participated in an affinity clustering exercise in which they grouped their ideas based on perceived similarities and differences. Detailed descriptions of the workshop activities are described in Table 1.

### Data collection: Phase 2

In phase 2, data were collected in May 2022 during PJL’s end of the school year symposium for principals of partner schools. Phase 2 focused on gathering feedback on the cluster analysis conducted in phase 1, validating findings, and voting on priority areas. Fifty-one principals from the partner schools from the 2021–2022 school year were invited to participate, and a total of 50 representatives from the schools attended the workshops, representing three regions of Madagascar. Two representatives from the national Ministry of Education also attended, however they were not involved in the voting process (Table 1).

### Data analysis

**Fig 3:**
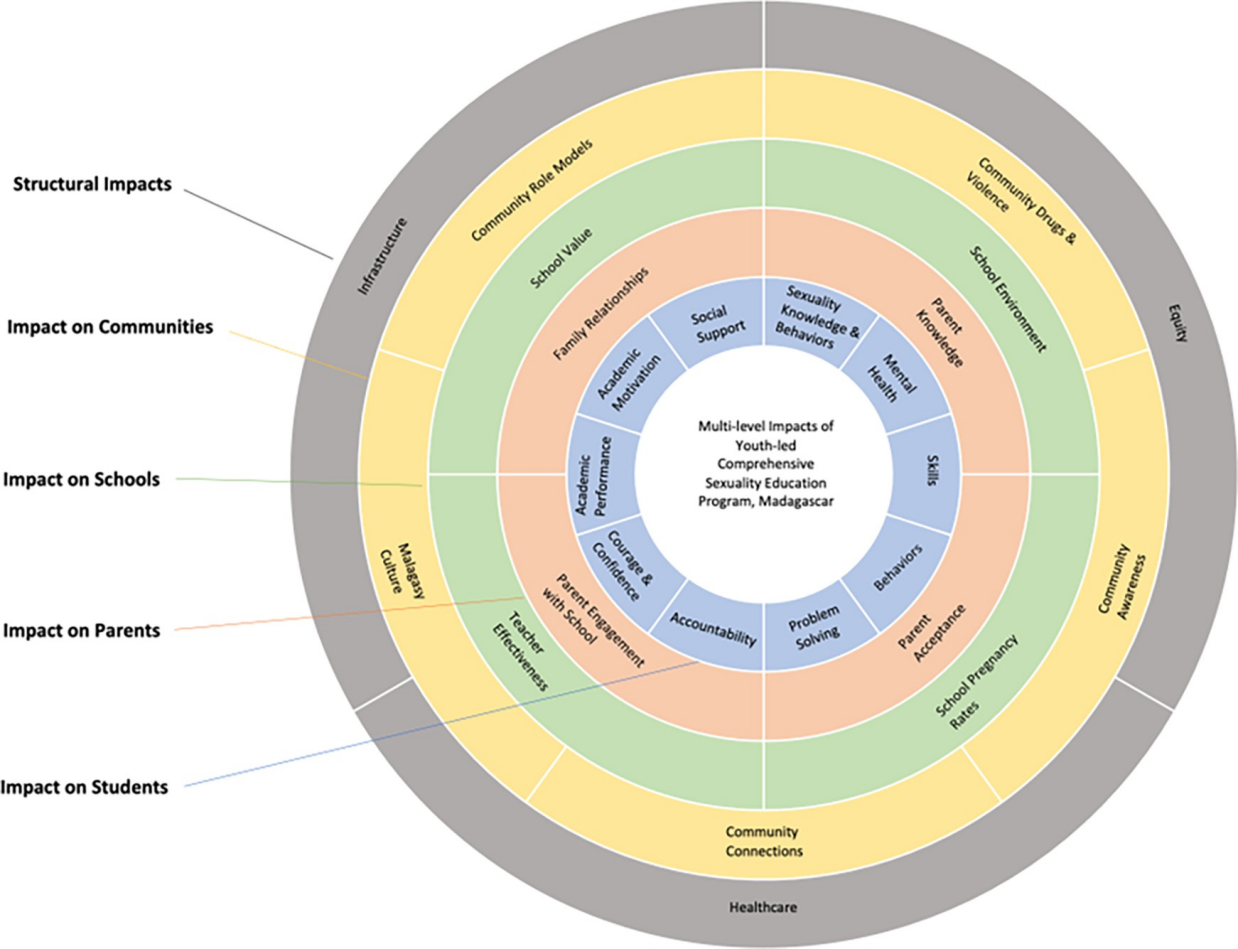
Multi-level impacts of youth-led comprehensive sexuality education model in Madagascar.

Two members of the research team consolidated key findings across the seven workshops conducted in phase 1, paying particular attention to key themes that were raised in multiple different workshops using qualitative content analysis. The team used grounded theory [41] to analyze the data using an inductive approach, in which initial cluster themes were compared across all workshops to draw broader comparisons, and the relationships between clusters were studied to propose a final conceptual model of the CSE program impacts (Fig 3). The final cluster labels were created by the team based on a review of the items within the clusters.

The tallied votes from the phase 2 “Visualize the Vote” exercise were collated in a standardized Microsoft excel spreadsheet. Basic counts were calculated for each cluster to reflect the principal’s observations of areas impacted by the CSE programming and the principal’s vote for the areas of impact most important to them. Translations of phase 2 principal feedback were reviewed by the coding team and select quotes were used as illustrative examples to contextualize the key clusters.

### Ethics

Permission to conduct this study was provided by PJL, the organization leading the CSE programming studied in this manuscript. Approval from an institutional review board was not obtained for the data collection since the intent of the workshops was to measure CSE program impact and is considered program evaluation. In advance of the workshop, a written invitation was sent to all potential participants explaining purpose of event. The team then followed up with a call to each school principal to confirm participation (or not) in the event and answer any questions they may have. Principals were not required to participate in the event and all participants were free to skip any activities and questions if they did not feel comfortable. Confidentiality was maintained as personal identifiers were removed from the dataset before analysis. Photographs are used with permission from participants.

### Inclusivity in global research

Additional information regarding the ethical, cultural, and scientific considerations specific to inclusivity in global research is included in the Supporting Information (S1 Questionnaire).

## Results

The study included 140 participants: 90 in phase 1, and 50 in phase 2. Participants shared their perceptions that the benefits of CSE programming impacts students’ lives in numerous ways, at multiple levels of impact, including a range of issues beyond direct sexuality knowledge and education. Results from the HCD workshops with school administrators illustrated the expansive reach of CSE programming into the lives of the students, their parents, their schools, and their communities. While we cannot conclude that these benefits are fully attributable to the program alone, the preliminary evidence presented here suggests that there are beneficial spillover effects of the CSE program on communities at large. We were also able to determine priority areas for continued and targeted investment in the scale up of the CSE model in Madagascar.

Fig 3 illustrates the various levels of impact of PJL’s CSE programming in Madagascar, which range from individual student impacts to broad community and structural impacts (Fig 3.). Table 2 includes specific items within each cluster/thematic area identified in phase 1, incorporates phase 2 input about the impacts most recognized/seen at PJL program schools, and identifies areas most important to address in programming moving forward, according to participants. No major differences were identified across the workshops nor by region.

**Table 2:** CSE program impacts on students, parents, schools, communities, and structures.

Impacts on Students	Participant Responses		
**Sexuality Knowledge & Behaviors**	Increases student knowledge about sexuality and confidence to speak about it		
** **	Increases student knowledge of changes during adolescence before they occur		
** **	Improves student knowledge and value of themselves and their bodies		
	Improves student ability to define types of relationships		
	Students start experimenting (with sex and love) †		
***Academic Motivation*** *	Increases student motivation to go to school and reduces dropouts		
** **	Increases student comfort at school		
** **	Increases student interest in academic studies		
** **	Increases student interest in reading		
** **	Makes lessons relevant to student’s everyday life		
	Increases student’s sense of pride to be part of PJL school		
	High interest in PJL programming leads students to be late to skip or neglect other classes †		
**Academic Performance **	Improves student test scores		
	Improves student’s ability to balance studies and entertainment/hobbies		
	Students distracted by PJL educator †		
***Courage & Confidence*** *	Increases student self-confidence, ability to take action, and fully participate		
** **	Increases student ability to share knowledge and ideas and opinions		
** **	Increases student courage to ask questions		
** **	Students have courage to share what they have learned in the community		
** **	Increases student bravery to disclose violence		
** **	Increases student ability to voice their problems		
** **	Students have misplaced courage †		
***Accountability*** *	Increases student responsibility		
** **	Promotes students looking to the future and goal setting		
** **	Develops students into leaders		
	Students become open minded		
**Skills**	Promotes student hobbies and uncovers hidden talents		
** **	Increases student general knowledge and skills		
** **	Improves student life skills		
**Behaviors **	Improves overall student behavior		
** **	Improves student hygiene behaviors		
	Students shift away from experimenting (with sex and love)		
**Problem Solving **	Provides students with supports and resources for problem solving		
	Addresses menstruation challenges for students		
**Mental Health**	Improves students’ management of emotions		
	Separating from the PJL educator makes the students sad †		
	PJL educators’ diversity of talents makes kids sad †		
***Social Support*** *	Increases solidarity and relationships between students		
** **	PJL students advise and help others		
	Strengthens the relationship between parents and children (students)		
	Students have a trustworthy person/resource to discuss personal matters		
	Students become attached to PJL educator †		
	Provides students and teachers with a mediator		
**Impact on Parents/Families**			
***Family Relationship ***		Strengthens the relationship between parents and children	
** **		Improves parenting	
** **		Fathers take responsibility	
** **		Parents with problems can use the PJL educator as a mediator	
**Parent Education & Training Opportunities **		Parents receive education	
** **		Parents become convinced and are motivated to attend trainings	
**Parent Engagement with School **		Parents allow their children (students) to always go to school (if they are doing PJL)	
		Encourages parent participation with school	
**Parent Acceptance**		Parents become open-minded about sexuality and taboo topics	
**Impact on Schools **			
***School Value ***		Develops and highlights value of the school	
** **		Surrounding schools start to ask for a partnership with PJL	
** **		School becomes a model school	
**School Environment **		Solidarity flourishes in the school	
** **		School becomes fun and welcoming due to PJL educator	
		Develops humanity	
** **		Students wish to become a PJL educator	
** **		Creates positive ambiance in the school	
** **		School becomes noisy/distracting †	
		School has a youth activist	
** **		School has an educator/colleague/helper	
** **		Fills a gap in school’s sexuality education program	
** **		Improves school management	
** **		Lack of financial support for additional resources (e.g., books, sports balls, games) †	
		Eliminates the gap between parents, and between parents and PJL educators	
***Teacher Effectiveness*** *		Increases ease of teaching lessons to students	
		Increases understanding and communication between teachers and students	
** **		Improves teaching methods and teachers’ skills	
** **		Teachers become comfortable to participate in all the different games	
** **		Difficulties coordinating the PJL work with the schedules of other teachers †	
** **		Improves interactions between students and teachers	
***School Pregnancy Rates*** *		Decreased rates of student pregnancy at school	
**Impact on Communities **			
**Community Role Models **			PJL educator is a role model/leader in the community
***Community Drugs & Violence ***			Facilitates the fight against drugs in the community
** **			Facilitates the fight against violence in the community
**Malagasy Culture **			Brings back Malagasy values/develops the culture in the community
** **			Erases taboos "little by little”
** **			Dance promoted by PJL contradicts local religious traditions †
**Community Connections**			Unites the surroundings, such as teachers and community members
			The community feels like it has friends who nurture and educate their children
** **			People trust the PJL educator
***Community Awareness*** *			The kids use the lessons/knowledge from the PJL educator in their community
** **			Transmits the knowledge gained from PJL and cares for community
** **			Develops the mentality of the community and comfort discussing sexuality
** **			Knowledge gained is transmitted to others in the community
** **			Community becomes interested in PJL
**Structural Impacts **			
**Equity**			Improves equity
**Healthcare**			Generates access to reliable healthcare
**Infrastructure**			Creates infrastructure and provides products (e.g., menstrual pads)

Italics = directly observed by school principals

* = identified as most important by school principals

† = may be interpreted as negative effects

### Program impact on students

The student-level impacts of the PJL CSE programming range from student sexuality knowledge and behaviors, to courage and confidence, to mental health and social support. One participant shared, “When students are self-confident, then they become brave to take responsibility, to talk about sexuality, to share ideas.” The CSE program also impacts behaviors beyond those just related to sexuality: “The [CSE] educator is a role model for kids, so those kids’ behavior improves because they are well-behaved.” The students develop strong relationships with the educators, leading them to have a trustworthy person to talk with and “they do not hide things and can get good advice, leading them to take care of themselves.”

While a majority of the discussion regarding the impact of the CSE program focused on positive outcomes, the participants did share select undesirable results from the programming. For example, some participants noted that high interest in the CSE programming leads students to be late to, skip, or neglect other classes. In addition, not all participants were convinced that the increased courage and confidence because of the CSE program is a positive thing and noted that sometimes students in the program have “misplaced courage,” leading to “talking back.”

During phase 2, principals identified student-level factors of academic motivation, courage and confidence, accountability, and social support as areas of impact that they have directly observed in their schools. They identified these same areas as those that are most important to them as principals. “The dropout rate has decreased since the PJL educator began,” said one principal. Another described why students are more engaged with school: "If another teacher doesn’t show up to work… they come knocking on the principal’s door asking if they can do [CSE program activities]. The CSE educator always has activities for the students. That’s what makes the students feel at-home at school.” According to another principal, “There are even students that say, ‘if it wasn’t for the educator, then I would have left school, but now I would like to become a PJL educator so I will stay!’”

### Program impact on parents

Participants expressed that the CSE program also impacts parents of students in the program. Specifically, it strengthens family relationships and dynamics between parents and children. One participant shared, “When parents are motivated to attend trainings then parents and children understand each other, and their relationship improves.” In addition, parents become engaged with the school, attend trainings, and become more open-minded about sexuality and taboo topics. Another principal spoke to the impacts of program on academic performance and enrollment from the parents’ perspective: “The success [passing] rate has increased. The kids have also started to follow the rules, as well as started to have big goals in life. As a result of the parents’ program, parents are encouraged to enroll their children at our school.”

During phase 2, the principals identified family relationships as an area of impact that they have directly observed but they did not identify it as one of the most important areas to them as principals.

### Program impact on schools

School participation in the PJL CSE programming is believed to add value to the school and contribute to a positive school environment. One group explained the relationship in this way: “Those students use the knowledge gained from the [PJL] educator and become role models. And when those students are role models, the school becomes a model school.” The programming also makes school a fun and welcoming place and fills a gap in the school’s sexuality education. One group noted “When students have complete education [including sexuality and humanities], then they are prepared for the future.”

In addition to impacting the school more generally, participants expressed that student participation in CSE impacts teacher effectiveness. Participants shared that the program makes it easier to teach students, increases communication between teachers and students, and improves teachers’ skills, as they often learn new tools and techniques from the PJL educators.

During phase 2, the principals identified school value, teacher effectiveness, and school pregnancy rates as three areas of impact that they have directly observed, and they identified these same areas as those that are most important to them as principals. Several principals explicitly addressed the impact of PJL’s CSE programming on school pregnancy rates. One shared, “At our school we used to see a lot of teenage pregnancies before, but since the PJL educator started, everyone has received advice since there is a lot of lessons about early pregnancy and help for the students and the teachers… now teenage pregnancies have stopped; we even have proof from the medical exams done during the physical portion of the middle school exit exam… there wasn’t a single pregnant 9^th^ grader.” Another school principal verified these impacts in their school as well: “It brings us joy to say that the pregnancy rate has been 0% during the three years the CSE educator has worked at our school. The concentration of the educator on lessons about sexuality are one reason for this, especially because they are practical…”

### Program impact on communities

Participants expressed that the CSE programming has spill-over effects in the community by creating community connections and awareness. For example, one participant expressed that the program “erases taboos little by little,” and another expressed that “the community feels like it has friends who nurture and educate their children.” Participants also expressed that the program facilitates the fight against domestic violence and substance use, and community members often turn to the PJL educators as a source of knowledge and trust in other aspects of life and community development.

During phase 2, the principals identified community drugs and violence and community awareness as two areas of impact that they have directly observed. The principals identified community awareness, specifically as it relates to community comfort discussing sexuality, as one of the most important areas to them as principals.

### Program impact on broader systems

In addition to addressing the impact that the CSE programming has on individuals, families, schools, and communities, participants shared that programming also effects structural issues including equity, healthcare, and infrastructure. For example, one participant said it “creates infrastructure and provides [menstrual] products.” One school monitor alluded to the fact that since the CSE programming is based in government schools, especially in rural areas, it provides support to otherwise neglected government schools. Even the poorest parents try to send their children to private schools; however, the PJL model appears to create an incentive for government schools and is a draw for students and their families: "All the students around want to move to a school with a PJL educator. I have a child who goes to a private school, my child always asks me every day what is going on with PJL. His friends always tell him what they do and learn with the PJL educator. My son asks me every day to transfer to the public middle school and I finally replied okay, next year I will put you in [that school].”

While the program demonstrated broader, structural impacts via the data collected in phase 1, they were not voted on as highest priority areas for the school principals and administrators in phase 2.

### Complexities of program impacts

While most spillover effects of the program were positive, it is important to note that participants did express cases of potential negative impacts of the program. These included “misplaced courage” as one participant described, in which students’ increased confidence led them to talk back to teachers and authority figures. In other rare cases, the program led the students to be “distracted” and created a noisy school environment, where some students lost interest in other classes after joining the CSE program. These negative impacts offer important lessons for the CSE program implementors, highlighting areas in which the CSE program can directly invest additional programming resources to lessen or mitigate such effects. For example, based on these findings, PJL now ensures that their educators use less noisy icebreakers to avoid disrupting other classes. They also co-developed, with the PJL educators, strategies on communicating their teaching methods with other teachers to foster greater understanding and synergies.

## Discussion

Our results align with the ongoing discourse regarding the broad reaching impacts that CSE can have, not only on individual students by improving sexual health knowledge and decision-making, but also on families, schools, and communities. The findings provide nuanced insight into the expansive impact of CSE programming on the lives of students, their parents, their schools, their communities, and broader structural issues.

In current scholarship and discourse in global CSE, experts state that CSE programming has a far-reaching impact much beyond individual level sexual health knowledge and behavior change [42]. Rollston argues, “While most people still believe sex education is just about sex, in reality it is so much more than that” [43]. For example, in a project called “Storymap” in the Netherlands, the creators aim to show the full impact that sex education programs have on communities and highlights the breadth of health indicators that are impacted by CSE [43]. Similarly, scholarship by Venwesenbeeck also suggests that CSE has wider positive impacts beyond individual students, including teacher-student relationships in the classroom, parent-child communication, and community norms; we witnessed similar findings in our Madagascar investigation [15]. Overall, our results align with current knowledge, that CSE can impact sexual health knowledge and decision-making, but can also have spill-over effects in other areas of students’ lives.

In terms of student level impacts, we found, expectedly, direct impacts on knowledge on sexuality education and reduced pregnancies among school-going students. However, we also witnessed several other effects that positively impact students’ lives, such as increases in self-confidence, academic performance, taking care of themselves, and improvements in overall behaviors. Similarly, another study summarizing findings over the course of 10 years from “The World Starts with Me” CSE program across 11 countries in Africa and Asia also found increases in self-confidence among students [15]. Some limited correlational evidence supports the qualitative findings generated in this study, that components of CSE increases social competencies, which can contribute to academic achievement, as well as reduced risk-taking and healthy relationships *[44]. The focus on developing social and emotional competencies within CSE have been linked to improved academic performance and mental and physical health outcomes [45]. A meta-analysis of studies of social and emotional learning (SEL) for students aged 5–18 years found that those SEL programs had improved social and emotional competencies, improved attitudes toward self, others and schools, and improved academic performance [45]. Our findings similarly suggest that proving holistic CSE, which uses youth-centered approaches, can increase students’ confidence and improve their behaviors both inside and outside the classroom.*

School-level impacts were also a key finding in our study, including improvements in teacher effectiveness and confidence among teachers as they learned new ideas to engage students in the classroom from CSE educators. Similarly, a study conducted in Pakistan found significant changes for teachers who participated in a CSE program and improvements in “teachers’ confidence, teaching skills, understanding of students’ issues, and relationships with students, as well as with their own families and communities” [15]. Thus, teachers can indirectly benefit from the effective implementation of a creative and engaging CSE program in their school, which is also likely to have spillover effects in their communities.

Furthermore, the Madagascar CSE program demonstrated perceived impacts on community issues, such as domestic violence and abuse, for which the CSE educators serve as a resource. Other studies also state that sex education can guide students in developing healthy self-identifies, and respectful relationships [43], which can help them to confront topics such as gender-based violence and intimate partner violence [46]. CSE programs teach that bullying is wrong and how to respond, but also get at the deep-rooted causes of bullying and violence, which are often linked to low-self-esteem [43]. By increasing self-esteem and confidence among students, CSE programming can help to decrease risk for students to bully and reduce conflict and violence at schools and in communities [47]. Additional literature suggests when schools educate about dating violence, and pair that with policies that aim to prevent the perpetration of violence, students are less likely to be victims of violence and sexual harassment on school property is significantly reduced [48].

While this community-engaged study has many strengths, especially centering the experiences and voices of those working directly with students in schools across Madagascar, there are several limitations to note. First, the participants in this study were limited to school principal, administrators, and teachers; future studies should consider including the voices and experiences of students and their parents regarding the impacts of the program. It should be noted that in some cases, school administrators are the most removed from the day-to-day lives of their students, and thus, teachers may have more concrete, first-hand experiences of the multi-level impacts of CSE. While this study provided clarity on the various ways that the CSE program is impacting communities, an understanding of the mechanisms explaining how this occurs was beyond the scope of this study. Future studies building upon this work should consider exploring the ways in which these impacts occur, addressing questions such as “How does CSE facilitate the fight against drugs and improve equity?” Importantly, the results presented are perceived benefits of participants, and future trials are needed to confirm these findings. Finally, the workshops were facilitated by a PJL team member, potentially introducing social desirability bias into participants’ responses. However, the PJL team has several dynamic accountability feedback mechanisms in place with school administrators, thus, administrators are accustomed to providing critical feedback to program staff with an understanding that it will be considered and used to improve the CSE program. We are confident that the final clusters and areas of impact of the CSE program presented here are contextually valid, especially since they were shared back to participants in phase 2 for confirmation. Future studies with representative samples are needed to explore how widespread these program impacts are, whether they are equally distributed across the whole community (e.g., are there people not reached by the program and why?), and how sustainable they may be. Finally, since this study focused on the discovery phase of HCD, future studies should consider building upon these findings and engage diverse stakeholders, including end-users, in ideation an implementation to directly build upon the opportunities outlined in this manuscript.

### Study implications

The findings uncovered in this participatory, community engaged HCD discovery study highlight specific areas in which CSE programming can invest additional resources and training to facilitate positive spillover effects of CSE. Notably, PJL has allocated additional resources for advocacy and partnership-building with the national Ministry of Education to present and discuss the multifaceted results of their CSE program. Building on their program’s impact on communities, PJL has also launched new cross-sectoral partnerships with protection-focused CSOs and government entities to continue to facilitate positive impacts of CSE in Madagascar.

## Conclusions

Comprehensive Sexuality Education (CSE) is a critical tool for addressing sexuality and reproductive health challenges among adolescents, including early pregnancy. Furthermore, our results highlight that CSE also has wide-reaching, multi-level impacts on students, schools, families, communities, and beyond. Human-centered Design (HCD) methods are particularly effective for engaging stakeholders in leadership roles to collaboratively explore program impacts and prioritize key areas for action.

## Supporting information

S1 Questionnaire Inclusivity in global health questionnaire. (DOCX)
